# The PILI@Work Program: a translation of the diabetes prevention program to Native Hawaiian-serving worksites in Hawai‘i

**DOI:** 10.1007/s13142-015-0383-3

**Published:** 2016-01-19

**Authors:** Claire K. M. Townsend, Robin E. S. Miyamoto, Mapuana Antonio, Guangxing Zhang, Diane Paloma, DeAnna Basques, Kathryn L. Braun, Joseph Keawe‘aimoku Kaholokula

**Affiliations:** Department of Native Hawaiian Health, John A Burns School of Medicine, University of Hawai‘i at Mānoa, 677 Ala Moana Blvd, Suite 1016, Honolulu, HI 96813 USA; Office of Biostatistics & Quantitative Health Sciences John A Burns School of Medicine, University of Hawai‘i at Mānoa, Honolulu, HI USA; Native Hawaiian Health Program, Queen’s Health Systems, Honolulu, HI USA; Office of Public Health Studies, University of Hawai‘i at Mānoa, Honolulu, HI USA

**Keywords:** Native Hawaiians, Worksite wellness interventions, Overweight/obesity, DPP translation

## Abstract

A previously translated Diabetes Prevention Program Lifestyle Intervention (DPP-LI) was adapted for delivery as a worksite-based intervention, called PILI@Work, to address obesity disparities in Native Hawaiians/Pacific Islanders. This study examined the effectiveness of PILI@Work and factors associated with weight loss at post-intervention. Overweight/obese employees of 15 Native Hawaiian-serving organizations received the 3-month component of PILI@Work. Assessments included weight, systolic/diastolic blood pressure, physical activity and functioning, fat intake, locus of weight control, social support, and self-efficacy. Weight, systolic/diastolic blood pressure, physical functioning, physical activity frequency, fat intake, family support, and eating self-efficacy improved from pre- to post-intervention. Regression analysis indicated that worksite type, decreased diastolic blood pressure, increased physical activity, and more internalized locus of weight control were significantly associated with 3-month weight loss. PILI@Work initiated weight loss in Native Hawaiians/Pacific Islanders. DPP-LI translated to worksite settings and tailored for specific populations can be effective for addressing obesity.

## INTRODUCTION

The prevalence of overweight and obesity in Native Hawaiians (72.5 %) and other Pacific Islanders (62.7 %) is among the highest of all ethnic groups in Hawai‘i [1, 2]. Coupled with this disparity in overweight/obesity are their higher burden of type 2 diabetes, cardiovascular disease, and other obesity-related diseases [3–7]. Evidence-based behavioral weight loss programs play an important role in reducing the prevalence of overweight/obesity and related health disparities [8, 9]. Equally important is the tailoring of these weight loss programs for specific ethno-cultural groups to increase program effectiveness, community receptiveness, and sustainability [10, 11].

The cultural adaptation of evidence-based interventions is viewed by many public health researchers and practitioners as integral in maintaining the effectiveness of these interventions when delivered to ethnic groups. Through these adaptations, evidence-based interventions are responsive and respectful of the specific knowledge and practices of the ethnic groups to whom the interventions are delivered [12]. In their review of the interventions to reduce diabetes and improve healthy eating and exercise, Barrera et al. found evidence that the cultural adaptation of evidence-based interventions assured or increased intervention effectiveness in specific cultural groups [13]. In another review, Bender and Clark found that interventions with greater cultural adaptations tended to be more appropriate and effective for ethnic groups, relative to interventions with moderate or minimal cultural adaptations [14].

An evidence-based program that has been effectively adapted to various contexts and settings is the Diabetes Prevention Program Lifestyle Intervention (DPP-LI) [15]. The DPP-LI was originally designed to be an intensive, 16-week, one-on-one lifestyle intervention that guides participants to modify their diet, physical activity, and other health behaviors to promote weight loss and its maintenance [16, 17]. Researchers and health professionals at the University of Pittsburgh Medical Center, along with the DPP intervention committee, developed the DPP-LI [17, 18]. Based on the social cognitive theory, DPP-LI utilizes evidence-based behavior change strategies including, but not limited to, identifying cues to action, regular monitoring of behaviors, goal setting, and cognitive restructuring [19, 20]. In a systematic review of 28 US-based studies across various settings and populations, DPP-LI translated programs produced a mean weight loss of 4 % of participants’ initial body weight [15]. A review of 17 studies on DPP-LI programs translated to African American communities found an overall weight loss rate of approximately 3 kg [21]. Participants in the original DPP-LI had an average age of 50.6 years (SD = 11.3), 68 % were female, and an average BMI of 33.9 (SD = 6.8) [22]. Only 5.3 % were Asian or Pacific Islanders, suggesting that the intervention lacked cultural tailoring specific to this group [22].

Utilizing a community-based participatory research (CBPR) approach, the Partnership for Improving Lifestyle Interventions (PILI) ‘Ohana Project (POP) translated the DPP-LI to Native Hawaiian and Pacific Islander communities [23]. The POP is a community-academic partnership that seeks to integrate community and scientific expertise to address health disparities among Native Hawaiians and other Pacific Islanders. The members of the POP during this translation included (1) Kōkua Kalihi Valley Family Comprehensive Services, a community health clinic; (2) Kalihi-Pālama Health Center, a community health clinic; (3) Ke Ola Mamo, Native Hawaiian Health Care System on O‘ahu; (4) Kula no na Po‘e Hawai‘i, a Hawaiian Homestead organization; and (5) Hawai‘i Maoli of the Association of Hawaiian Civic Clubs.

The DPP-LI translation was informed by data collected from 333 Native Hawaiian and other Pacific Islander community leaders, members, and stakeholders through interviews and focus groups, as described in detail by Mau et al. [24]. Specifically, each of the five partnering organization conducted 3 focus groups and 3 key informant interviews, for a total of 15 focus groups and 15 key informant interviews. Focus group participants were recruited using community flyers and newsletters and were asked questions on personal motivation to lose weight, the influence of family, friends, and community on individual weight loss, and thoughts on addressing overweight/obesity in their communities. Key informants were asked how overweight/obesity is impacting their community, resources, or lack thereof, in their community to address overweight/obesity, and thoughts on how to address overweight/obesity. Additionally, a community “windshield tour” (i.e., walking/driving through a specific community) was done in various Native Hawaiian and Pacific Islander communities to visually survey the physical activity (e.g., parks and walking trails) and healthy eating infrastructure and note its condition and utilization [30].

The adapted weight loss intervention (PILI Lifestyle Program; PLP) served as the first 3 months of a larger 9-month intervention (the latter 6 months provided lessons in weight loss maintenance). In the initial pilot testing of the 3-month PLP (*N* = 169) in POP member organizations, it was found to lead to significant, albeit modest, improvement in weight loss (−1.5 kg, SD = 3.5), improvements in both systolic blood pressure (−6.0 mmHg, SD = 18) and diastolic blood pressure (−2.8 mmHg, SD = 11), and an increase in distance walked in 6 min (a measure of physical functioning; 42 ft, SD = 124) [24]. Further testing in partnering communities yielded similar results: improvements in weight loss (−1.7 kg, SD = 3.5), systolic blood pressure (−3.3 mmHg, SD = 18.6), diastolic blood pressure (−3.4 mmHg, SD = 12.5), and distance walked in 6 min (106.6 ft, SD = 238.4) [25].

Worksites offer a promising venue for delivering lifestyle interventions, such as the adapted PLP [26]. The opportunities for group-based interactions, environmental modifications, and support from colleagues make workplaces natural settings for such programs [27]. Worksite characteristics that may increase the effectiveness of healthy lifestyle programs include social support and opportunities to modify the environment through policy (e.g., providing healthier food options or access to physical activity facilities) [28–32]. In a systematic review conducted by Whittemore et al., the effectiveness of the DPP was evaluated based on intervention setting [33]. DPPs implemented in worksites and churches were found to have the greatest potential of reaching a diverse sample of adults when compared to DPPs implemented in clinics, hospitals, and other community settings [33]. Average weight loss in these sites was 3.17 kg at 12 month assessment [33].

To increase the reach of the PLP and test its effectiveness in worksite settings, partnerships were established between the Department of Native Hawaiian Health at the John A. Burns School of Medicine at the University of Hawai‘i at Mānoa and 14 Native Hawaiian-serving organizations (15 organizations including the medical school, 22 cohorts) who expressed an interest in health promotion programs. Through these partnerships, university and worksite-based researchers implemented the PLP, renamed PILI@Work, as a worksite wellness program. The aims of this paper are to examine the effectiveness in translating the Diabetes Prevention Program in Native Hawaiian-serving worksites and to elucidate the socio-demographic, psychosocial, behavioral, and biological factors associated with weight loss efforts among four types of worksite settings.

## METHODS

### Worksite partners and participants

‘Imi Hale Native Hawaiian Cancer Network, funded through the National Cancer Institute’s Community Networks Program Centers (CNPC) initiative, supported this study. The CNPC initiative was designed to address cancer disparities in ethnic minorities by engaging communities in research, and the ‘Imi Hale CNPC focused on reducing cancer health disparities experienced by Native Hawaiians in Hawai‘i [34, 35].

Participants were recruited from the employees of 15 Native Hawaiian-serving organizations (i.e., organizations with a mission to serve Native Hawaiian communities), resulting in 22 cohorts and a total of 275 enrolled participants. Native Hawaiian-serving organizations attract a large number of Native Hawaiian and Pacific Islander employees. By focusing on these organizations, the study was able to recruit a large number of Native Hawaiian and Pacific Islanders. The 15 worksites are grouped as social service organizations (SS; 8 cohorts), health centers (HC; 6 cohorts), Native Hawaiian Health Care Systems (NHHCS; 3 sites), and academic institutions (AI; 5 sites). These worksites are located across the State of Hawai‘i, with at least one participating worksite on each of the six major islands O‘ahu (9), Maui (1), Lāna‘i (1), Moloka‘i (2), Kaua‘i (2), and Hawai‘i Island (2). The islands differ in resources and urbanization. For instance, O‘ahu has just under 1,000,000 residents while Lana‘i has only 3000.

In addition to being an employee of the worksite, the eligibility criteria for participation in the PILI@Work weight loss intervention included (a) at least 18 years old, (b) overweight or obese based on WHO recommendations (BMI ≥ 25 for Caucasians, Native Hawaiians or Pacific Islanders or ≥ 23 for individuals of Asian ancestry), and (c) willing and able to fully participate in the program to include healthy eating and calorie control and 150 min of brisk walking, or the equivalent, per week. Individuals who planned to leave the organization or community during the intervention period, were or became pregnant, had any dietary/exercise restrictions or limitations and/or co-morbid conditions that would have precluded them from fully participating were excluded from participation.

Participant recruitment and retention strategies were culturally informed and emphasized trust and worksite involvement. Implementation and assessment of the intervention were carried out by trained community and academic researchers, and overall intervention approval was given by Institutional Review Boards (IRB) at the University of Hawai‘i, the Native Hawaiian Health Care Systems, and the Queen’s Health Systems. This study was registered with Clinicaltrials.gov, number NCT01652989.

### Intervention

The adapted DPP-LI was part of a larger 9-month weight loss maintenance intervention called the PILI Lifestyle Program (PLP). The eight lessons of this adapted 3-month DPP-LI, the lesson-based activities, and the corresponding DPP-LI session are presented in Table 1. A detailed description of the PLP is provided by Kaholokula et al. [25]. Despite the shortened delivery schedule (i.e., eight lessons delivered over 12 weeks), the PLP retained all original foci and strategies from the DPP-LI, as shown in Table 1. The cultural adaptations were done based on the results of the community need assessment, briefly described in the introduction, and on the input of the PILI ‘Ohana Project Intervention Steering Committee [24]. The lessons were adapted to be delivered in group settings of 10–20 people. This was done both for feasibility reasons and to capitalize on the Hawaiian cultural concept of ‘ohana (i.e., interdependence and the preference for working in groups). The name of the intervention, PILI ‘Ohana, also uses this concept as “pili” in Hawaiian means to stick or adhere to. New lessons on economical healthy eating and doctor-patient communication were created based on themes identified in the assessments. Each session lasted about 1 h and was designed to be interactive between facilitator and participants and among participants via in-session activities. The first four lessons were delivered weekly and remaining four lessons were delivered every other week, for a total for 3 months.

**Table 1:** Summary of the 3-month PILI lifestyle program (PLP)

Lessons	Topic	Lesson-based activities	Corresponding DPP-LI sessions
1 (Mo 1)	Intro to PILI Lifestyle Program Slippery Slope of Lifestyle Change Ways to Stay Motivated	Passport to Health Booklet Daily Food Diary/Nutrition Log My Progress Chart	1A: Welcome to the Lifestyle Balance Program 12: The Slippery Slope of Lifestyle Change 16: Ways to Stay Motivated
2 (Mo 1)	Getting Started Three Ways to Eat Less Fat Being Active: A Way of Life	Keep It Safe Brochure Safe & Easy Exercises Brochure Menu Makeover	1B: Getting Started Being Active 3: Being Active: A way of Life 5: Three Ways to Eat Less Fat
3 (Mo 1)	Get Moving Be a Fat Detective Move Those Muscles	Cooking Light Brochure My Food Label/Nutrition Labels Lower Fat in Meats	1B: Getting started being active and Getting Started Losing Weight 4: Be a Fat Detective 2: Move Those Muscles
4 (Mo 1)	Making it Fun Healthy Eating The 4 Keys to Healthy Eating Out Starting Your Activity Plan	Eating Out Brochure Free Foods Social Gatherings Brochure F.I.T.T. Plan	6: Healthy Eating 10: Four Keys to Health Eating Out 13: Jump Start Your Activity Plan
5 (Mo 2)	Keeping it Going Tip the Calorie Balance Economics of Healthy Eating	My Grocery List Write-in Card PILI Grocery Checklist Card Eat Smart. Eat Fresh. Brochure	8: Tip the Calorie Balance
6 (Mo 2)	Taking Charge Take Charge of What’s Around You Make Social Cues Work for You	Options Wallet Card	7: Take Charge of What’s Around You 14: Make Social Cues Work for You
7 (Mo 3)	Talking it Out Effective communication with MD Problem Solving Skills	Lifestyle Balance Problem Solver Roadblocks Brochure Talk With Doc Handout	9: Problem Solving
8 (Mo 3)	Wrapping it Up Talk Back to Negative Thoughts You Can Manage Stress Wrap Up—Discuss Next Phase	None	11: Talk Back to Negative Thoughts 15: You Can Manage Stress

The PLP as delivered in the worksite was renamed PILI@Work. Minimal modifications (e.g., references to the delivery site, length of intervention) were made to the PLP for worksite delivery. Trained worksite peer facilitators delivered the intervention. The PILI@Work facilitators were trained in delivery of the curriculum, behavior change strategies, motivational interviewing, and strategies for incorporating examples and activities specific to their organization. The intervention was delivered in the actual work setting and occurred either during an extended lunch period or immediately after work hours. Most of the facilitators were employees of the organization (referred to as internal facilitators, *n* = 15) while several were hired by the organization or brought in by the PILI@Work Intervention Steering Committee (ISC) specifically to facilitate the intervention (i.e., referred to as external facilitators, *n* = 7).

The study population for the PLP included Native Hawaiians or Other Pacific Islanders (including Filipino) ≥18 years old, with a BMI ≥ 25 kg/m^2^ (≥23 kg/m^2^ for those of Filipino ancestry). In comparison, the study population for the original DPP-LI included individuals ≥ 25 years old with a BMI ≥ 24 kg/m^2^, fasting plasma glucose of 95 to 125 mg/dL and a plasma glucose value of 140 to 199 mg/dL 2 h after a 75-g glucose load.

### Study design and procedures

The study used a CBPR approach that allowed the worksite and academic partners to work collaboratively. The ISC was formed during the development of the grant proposal and included members on the managerial or executive leadership teams of the participating worksites as well as the Principal Investigators and research project associates from the University of Hawai‘i and ‘Imi Hale Native Hawaiian Cancer Network. However, during the course of the project, the composition of the ISC changed as new organizations became interested in the project and two of the original members were unable to participate, due to their own organizational constraints. The ISC provided guidance and leadership for implementing the PLP curriculum in Native Hawaiian-serving organizations. Additionally, the ISC established a “CBPR Principles and Guidelines for Overall Governance,” which described the project’s shared mission, guiding principles, roles, and responsibilities of worksite personnel involved in this study and the academic partners, the decision-making process, the management and dissemination of data, and the evaluation protocols of the CBPR partnership. Two of the ISC members are co-authors on this paper, which was sent to all ISC members for input prior to publication.

After obtaining consent, all eligible participants were enrolled in the PILI@Work intervention. This study used a pre- and post-intervention evaluation design. Participants were assessed at baseline (i.e., within 2 weeks before beginning the intervention) and at 3-month follow-up (i.e., within 2 weeks of completion of the 3-month intervention).

### Assessment instruments

Assessment instruments were selected for their validity, sensitivity to change, literacy level, ease of administration, cultural appropriateness, and relevance to the study intervention and target population. The assessments occurred at the worksites and followed a standardized protocol as described in previous publications [4, 24, 25, 36].

#### Clinical measures

Clinical measures included body weight (kg), measured with an electronic scale (Tanita BWB800AS); height (cm), collected using a stadiometer (Seca 222 stadiometer); and blood pressure (systolic and diastolic), taken with an electronic blood pressure device (HEM-907XL IntelliSense automatic blood pressure device). All clinical measurements were done in a private location at the assessment site, away from other study participants to protect participant confidentiality. These measurements were collected twice, and the averages were used as the final measure for this study.

#### Socio-demographics

Date of birth, sex, marital status, education level, and self-reported ethnic group data were collected. The ethnic groups included Native Hawaiians (i.e., the indigenous people of Hawai‘i), other Pacific Islanders (i.e., people with origins in the original inhabitants of Polynesia including Filipinos), Asians (e.g., Japanese, Chinese, Korean), and “other” (e.g., African American, Latino).

#### Physical functioning

The physical functioning of participants was evaluated by using a 6-min walk test (6MWT). A standardized script containing instructions for the participant was read aloud by research staff before and during the walk test based on the American Thoracic Society’s 6MWT guidelines. Participants were instructed to walk as far as possible around a 30 ft course, for 6 min without running [37]. If necessary, participants were permitted to stop and rest and could resume walking when they were able. The number of laps and overall distance walked were recorded and converted to total feet walked for analysis.

#### Exercise frequency

Frequency of moderate and vigorous physical activity and exercise and the subsequent change in activity levels were assessed using a 3-item self-report Physical Activity Questionnaire [38]. The 2 items included assessment of moderate physical activity, rated by frequency on a response scale of 1 (more than 4 times/week) to 5 (rarely or never) and assessment of vigorous physical activity, rated by frequency on a response scale of 1 (more than 4 times/week) to 5 (rarely or never). Responses to the items were averaged to calculate total exercise frequency.

#### Fat intake

Dietary fat intake was assessed using a 39-item modified version of the Eating Habits Questionnaire [39]. The questionnaire assesses fat consumption in four categories: (1) modification of meat, (2) avoidance of fat, (3) modification/substitution of fatty foods, and (4) replacing fatty foods with vegetables. Items are scored using a 4-point response scale ranging from 1 (always) to 4 (never). Adding the mean fat consumption scores for each category and dividing it by 4 obtained the summary score. An overall summary score of ≥2.5 indicates fat comprises more than 30 % of calories in a participant’s diet.

#### Locus of weight control

Locus of weight control was measured using the Weight Locus of Control Scale (WLOC) [40]. This scale assesses a participant’s belief in how their weight is controlled—how much their weight is controlled by them (internal locus of control) vs. other factors (external locus of control). The WLOC is a 4-item survey scored on a Likert-type response scale from 1 (strongly disagree) to 5 (strongly agree). Two of the items are internally focused (e.g., whether I gain, lose, or maintain my weight is entirely up to me) and two are externally focused (e.g., being the right weight is largely a matter of good fortune). Possible range of total scores is from 4 to 20, with 4 indicating extreme externality and 20 indicating extreme internality.

#### Exercise self-efficacy

Exercise self-efficacy was assessed using the Self-Efficacy for Exercise Scale (SEE) [41]. This scale measures a participant’s self-efficacy expectations related to their ability to continue to exercise despite encountering barriers. The SEE is a 9-item scale that asks a participant to rate his or her confidence to engage in 60 min of exercise per week given the situation described in the nine items. Confidence is rated on a response scale from 1 (not sure at all) to 5 (completely sure). The SEE is scored by adding the ratings and dividing by the number of ratings completed. Scores range from one to five with higher scores indicating greater exercise self-efficacy.

#### Eating self-efficacy

A modified Eating Self-Efficacy Scale was used to measure the participants’ self-efficacy expectation related to their ability to control their eating in potentially difficult situations [42]. The ESE is a 9-item scale, which asks participants to rate how confident they are that they could resist eating in nine different situations (e.g., when watching TV, when high-fat foods are available, when tired or stressed out). Confidence is rated on a response scale from 1 (not sure at all) to 5 (completely sure). The ESE is scored by adding the ratings and dividing by the number of ratings completed. Scores range from one to five with higher scores indicating greater eating self-efficacy.

#### Family and community support

Perceived family and community support were assessed using the Family Support Scale (FSS) and the Community Support Scale (CSS) [43]. These two scales measure participants’ perceptions of family support (6 items) and community support/resources (5 items) in helping to achieve and maintain healthy eating and exercise. Participants are asked to indicate how much support they are receiving, or not receiving, for each item. An example of a family support item is “my family encourages me to lose weight.” An example of a community support item is “my community is safe for me to walk around or exercise in.” Items are scored on a 5-point Likert scale, from 1 (never) to 5 (very often). Both scales are scored by adding the ratings and dividing by the number of ratings completed. Scores range from one to five with higher scores indicating greater family or community support.

### Statistical analysis

Descriptive statistics were calculated first to summarize demographic variables and pre- and post-intervention clinical, psychosocial, and behavioral variables. Frequencies and percentages were calculated for the categorical variables (e.g., ethnicity). Means and standard deviations for continuous variables (e.g., age and weight) were calculated. Significant differences by worksite type were determined using Chi-square (*χ*^2^) analyses for categorical variables and one-way ANOVA for continuous variables. Tukey-Kramer post hoc analyses were done to detect significant differences across four worksites for continuous variables. Paired *t* tests were used to examine changes in clinical, psychosocial, and behavioral measures by worksite type and for the combined sample from pre-intervention to post-intervention. Changes at post-intervention were calculated as 3-month assessment value minus baseline assessment value. One-way ANOVA was used to compare the differences in the changes across the worksite types. If a significant difference was found, this was followed by a Tukey-Kramer procedure to determine how the worksite types differed. The bivariate association of demographic variables at baseline and clinical, psychosocial, and behavioral change variables was examined using correlation analysis. A multivariable linear model was developed to identify demographic, clinical, psychosocial, and behavioral variables contributing to weight loss at 3-months. Included in this model were the demographic variables and specific change variables which had a significant (i.e., *p* < 0.05) bivariate association with weight change at 3-months. All statistical analyses were two-sided and performed using SAS software version 9.4 (SAS Institute Inc., Cary, NC). An alpha level of 0.05 was used to determine statistical significance.

## RESULTS

### Baseline characteristics

Of the 275 people who were recruited and participated in baseline assessments, 217 completed the 3-month assessment for a retention rate of 78.9 %. This rate varied significantly (*p* = 0.008) across the four worksite groups as follows: AI (69.9 %), HC (73.6 %), NHHCS (88.6 %), and SS (88.8 %). Baseline characteristic were comparable between completers (*n* = 217) and dropouts subjects (*n* = 58), with *p* values >0.14. The number of lessons received was an exception with completers receiving 6.2 (SD = 2.08) compared to dropouts receiving 2.1 (SD = 2.09; *p* < 0.001). The number of lessons received also varied significantly by worksite group. HC participants received the most lessons (*M* = 7.35, SD = 1.28), followed by NNHCS (*M* = 6.61, SD = 1.69) and SS (*M* = 6.03, SD = 1.93) with AI participants receiving the fewest (*M* = 4.78, SD = 2.42).

Baseline characteristics for each worksite type and the combined sample (*n* = 217) are summarized in Table 2. Participants had a mean age of 46.2 years (SD = 11.3) and BMI of 32.9 (SD = 6.9). Over one third (38.3 %) were Native Hawaiian, 21.2 % were Other Pacific Islander, 21.2 % were Asian, and 13.8 % were Caucasian. The majority were females (87.1 %). There were significant differences at baseline among worksite types on weight (*R*^2^ = 0.044, *F*_(3,213)_ = 3.31, *p* = 0.021) and BMI (*R*^2^ = 0.044, *F*_(3,213)_ = 3.28, *p* = 0.022). Participants at NHHCS and SS were heavier at baseline (89.0 and 92.1 kg, respectively). Additionally, there were baseline differences in ethnic groups (*χ*^2^_(12,209)_ = 48.55, *p* < 0.0001) and education level (*χ*^2^_(9,205)_ = 25.39, *p* = 0.003).

**Table 2:** Participants’ baseline socio-demographic characteristics, clinical measures, and behavioral measures by community site and combined sample

Characteristics	Worksite group					Combined total
	Academic *n* = 51	HC *n* = 64	NHHCS *n* = 31	SS *n* = 71	*p* value	*n* = 217
Facilitator type					0.053	
Internal	27 (52.94)	49 (76.56)	21 (67.74)	43 (60.56)		140 (64.52)
External	24 (47.06)	15 (23.44)	10 (32.26)	28 (39.44)		77 (35.48)
Ethnicity					<0.001	
Asian	16 (31.37)	17 (26.56)	5 (16.13)	8 (11.27)		46 (21.20)
Caucasian	12 (23.53)	14 (21.88)	3 (9.68)	1 (1.41)		30 (13.82)
Native Hawaiian	12 (23.53)	12 (18.75)	14 (45.16)	45 (63.38)		83 (38.25)
Other Pacific Islander	9 (17.65)	12 (18.75)	9 (39.03)	16 (22.54)		46 (21.20)
Other	0 (0.00)	3 (4.69)	0 (0.00)	1 (1.41)		4 (1.84)
Age, years	46.69 (10.87)	45.89 (10.56)	46.28 (12.95)	46.11 (11.73)	0.985	46.21 (11.30)
Females	43 (84.31)	58 (90.63)	27 (87.10)	61 (85.92)	0.765	189 (87.10)
Education level					0.003	
≤High school/GED	1 (1.96)	3 (4.69)	4 (12.90)	5 (7.04)		13 (5.99)
Some college/tech	5 (9.80)	13 (20.31)	8 (25.81)	23 (32.39)		49 (22.58)
College degree	21 (41.18)	25 (39.06)	14 (45.16)	15 (21.13)		75 (34.56)
Graduate/professional	23 (45.10)	16 (25.00)	3 (9.68)	26 (36.62)		68 (31.34)
Marital status					0.969	
Never married	12 (23.53)	11 (17.19)	8 (25.81)	15 (21.13)		46 (21.20)
Currently married	30 (58.82)	35 (54.69)	17 (54.84)	44 (61.97)		126 (58.06)
Interrupt marital status	8 (15.69)	11 (17.19)	4 (12.90)	10 (14.08)		33 (15.21)
Height (cm)	162.62 (8.97)	160.72 (7.73)	160.81 (7.84)	162.85 (8.71)	0.382	161.87 (8.38)
Weight (kg)	84.41 (19.89)	81.09 (14.47)	88.98 (23.83)	92.09 (25.81)	0.021	86.60 (21.64)
Body mass index (kg/m^2^)	31.67 (5.85)	31.38 (5.17)	34.16 (7.61)	34.50 (8.25)	0.022	32.86 (6.92)
Systolic blood pressure (mmHg)	125.74 (16.78)	122.20 (15.58)	126.82 (16.46)	126.68 (79.82)	0.403	125.17 (16.68)
Diastolic blood pressure (mmHg)	79.22 (10.84)	78.87 (10.29)	80.56 (9.45)	79.82 (11.43)	0.889	79.51 (10.64)
6-min walk test (ft)	1413.38 (186.03)	1437.49 (161.25)	1373.97 (199.36)	1376.15 (218.07)	0.242	1402.77 (193.05)
Physical activity frequency score	3.49 (1.16)	3.66 (1.09)	3.19 (1.27)	3.56 (0.97)	0.278	3.52 (1.10)
Fat in diet score	2.70 (0.41)	2.77 (0.35)	2.85 (0.45)	2.78 (0.40)	0.407	2.77 (0.39)
Family support scale	21.71 (3.81)	21.65 (4.02)	21.96 (4.81)	20.81 (4.62)	0.563	21.43 (4.30)
Community support scale	20.98 (3.25)	21.02 (3.30)	21.54 (2.66)	20.27 (3.21)	0.320	20.83 (3.18)
Locus of weight control	17.14 (2.47)	16.75 (2.19)	16.50 (2.34)	16.78 (2.45)	0.723	16.81 (2.35)
Self-efficacy for exercise	3.37 (0.90)	3.28 (0.94)	3.68 (0.91)	3.25 (0.89)	0.249	3.34 (0.91)
Eating self-efficacy	3.21 (0.99)	3.08 (0.94)	2.94 (0.88)	3.11 (0.95)	0.725	3.10 (0.94)
Number of lessons	4.78 (2.42)	7.35 (1.28)	6.61 (1.69)	6.03 (1.93)	<0.001	6.20 (2.08)

Data shown *M* (SD) or *n* (%). Column numbers may not be equal to 100 % due to round-off or unknown values. *p* value for categorical variable based on Fisher’s exact or Chi-sq test; *p* value for continuous variable based on one-way ANOVA. Fat in diet score was calculated using available case analysis instead of complete case analysis, in terms of handling partly missing sub-scores. Number of lessons taken between baseline and 3-month visits, range = 0 to 8

### Change in clinical and behavioral measures

Table 3 summarizes the changes from pre- to post-intervention assessments for the combined data of the four worksite groups. Combined, there were significant improvements in weight (*M* = -1.2 kg, SD = 2.6; *t*_(216)_ = −6.65, *p <* 0.001) and systolic BP (M = -2.8 mmHg, SD *=* 12.5; *t*(215) = −3.29, *p =* 0.001) and diastolic BP (*M* = -2.0 mmHg, SD *=* 8.1; *t*_(215)_ = −3.67, *p <* 0.001). There were also significant improvements in feet walked during the 6MWT (*M* = 69.4 ft, SD *=* 136.3; *t*_(209)_ = 7.38, *p <* 0.001), physical activity frequency (*M =* -0.6, SD *=* 1.1, *t*_(216)_ = −7.59, *p* < 0.001), and fat in diet (*M =* -0.2, SD *=* 0.3, *t*_(216)_ = −8.96, *p <* 0.001). Other statistically significant changes included an increase in perception of family support (*M* = 0.7, SD *=* 3.0; *t*_(169)_ = 2.88, *p* = 0.004), a decline in perception of community support (*M =* -0.6, SD *=* 2.8, *t*_(168)_ = −2.62, *p* = 0.010), and an increase in eating self-efficacy (*M =* 0.1, SD *=* 0.8, *t*_(168)_ = 2.18, *p =* 0.031).

**Table 3:** Pre- to post-intervention changes in clinical and behavioral measures in combined sample

Characteristics	Worksite group				Combined total	*p* value
	Academic	HC	NHHCS	SS		
Weight (kg)*	−0.75 ± 2.07	−1.72 ± 2.9	−1.84 ± 2.9	−0.73 ± 2.51	−1.18 ± 2.63	<0.001
Body mass index (kg/m^2^)*	−0.3 ± 0.75	−0.66 ± 1.08	−0.71 ± 1.1	−0.27 ± 0.91	−0.45 ± 0.97	<0.001
Systolic blood pressure (mmHg)	−3.03 ± 11.54	0.97 ± 11.11	−4.81 ± 13.83	−5.11 ± 13.27	−2.8 ± 12.53	0.001
Diastolic blood pressure (mmHg)	−0.98 ± 7.2	−1.48 ± 7.37	−2.19 ± 8.18	−3.14 ± 9.1	−2.01 ± 8.05	<0.001
6-min walk test (ft)	87.45 ± 140.51	53.68 ± 102	43.84 ± 132.13	81.3 ± 159.38	74.65 ± 154.71	<0.001
Physical activity intensity score	−0.44 ± 1.15	−0.88 ± 0.98	−0.42 ± 1.07	−0.43 ± 1.12	−0.57 ± 1.1	<0.001
Fat in diet score	−0.19 ± 0.28	−0.21 ± 0.27	−0.21 ± 0.36	−0.19 ± 0.38	−0.19 ± 0.33	<0.001
Family support scale	1 ± 3.13	0.78 ± 2.88	0.08 ± 2.93	0.54 ± 2.94	0.71 ± 2.97	0.002
Community support scale*	−1.73 ± 3.18	−0.16 ± 2.54	−0.58 ± 2.86	−0.18 ± 2.58	−0.56 ± 2.79	0.010
Locus of weight control	−0.26 ± 1.8	0.47 ± 1.81	0.04 ± 1.49	−0.12 ± 2.13	0.05 ± 1.88	0.717
Self-efficacy for exercise	−0.12 ± 0.71	0.04 ± 1.13	−0.59 ± 1.37	−0.06 ± 0.97	−0.12 ± 1.04	0.143
Eating self-efficacy	0.06 ± 0.7	0.16 ± 0.84	0.13 ± 0.91	0.16 ± 0.82	0.15 ± 0.81	0.020

For the combined total or within community, analysis based on paired *t* test (H0: μpair-diff = 0). Data are shown as mean ± standard deviation. Refer to Table 2 for baseline means and standard deviation. *BP* blood pressure, *6MWT* 6-min walk test, *Physical Activity Fq* physical activity frequency score. Across four communities, one-way ANOVA used to compare the changes from baseline. Differences are indicated by an asterisk next to the name of the measure, adjusted for differences at baseline﻿﻿﻿﻿﻿. Change scores based on post-intervention values minus baseline values. Physical Activity Frequency Score: frequency of moderate-vigorous physical activity, range 1 = ≥4 times/week (more active) to 4 = rarely or never (less active). Thus, lower scores are more active and a negative change means more physical activity. Fat in Diet Score of 2.5 or greater indicates greater than 30 % of calories from fat.

Differences in outcomes at 3-months were further examined by worksite group, adjusting for their baseline values. Across the four worksite groups, outcomes in diastolic and systolic blood pressure, physical functioning, fat in diet, family support, locus of weight control and eating and exercise self-efficacy did not vary significantly. However, significant differences between worksite groups were found in weight (*R*^2^ = 0.043, *F*_(4,212)_ = 2.99, *p* = 0.032), BMI (*R*^2^ = 0.043, *F*_(4,212)_ = 3.11, *p* = 0.027), and perceived community support (*R*^2^ = 0.124, *F*_(4,164)_ = 2.92, *p* = 0.036). HC (−1.72 kg, SD = 2.90, *t*_(63)_ = −4.75, *p* < 0.001) and NHHCS (−1.84 kg, SD = 2.90, *t*_(30)_ = −3.55, *p* = 0.001) employees had greater weight loss than AI (−0.75 kg, SD = 2.07, *t*_(50)_ = −2.58, *p* = 0.013) and SS (−0.73 kg, SD = 2.51, *t*_(70)_ = −2.45, *p* = 0.017). Only SS employees had significant improvements in systolic (−5.11 mmHg, SD = 13.27, *t*(_70)_ = −3.25, *p* = 0.002) and diastolic (−3.14 mmHg, SD = 9.10, *t*_(70)_ = −2.91, *p* = 0.005) blood pressure. Only AI employees had a significant decrease in perceived community support (−1.73, SD = 3.18, *t*_(36)_ = −3.26, *p* = 0.002).

Difference in weight loss at 3-months was also examined by ethnic group. The data were divided into two different groupings: (1) four categories: White, Native Hawaiian, Other Pacific Islander, and Asian and (2) three categories: White, Asian, and Native Hawaiian, and Other Pacific Islander. Controlling for worksite group, neither of these groupings yielded significant differences in weight loss: for four categories of ethnicity (*F*_(3,197)_ = 0.02, *p* = 0.995) and for three categories of ethnicity *F*_(2,198)_ = 0.03, *p* = 0.967).

### Bivariate analyses

The bivariate associations between baseline socio-demographic and clinical characteristics and weight loss at 3-month assessment and between change in clinical, psychosocial, and behavioral variables and weight loss at 3-months were examined (data not shown in tables). No significant association between socio-demographic or baseline clinical characteristics and weight loss was found. However, decrease in diastolic blood pressure (*r* = 0.174, *p* = 0.010), increase in physical activity (*r* = 0.224, *p <* 0.001), decrease in fat in diet (*r* = 0.149, *p* = 0.029), decrease in community support (*r* = −0.238, *p* = 0.002), and more internal locus of weight control (*r* = −0.223, *p* = 0.003) were all significantly associated with weight loss at 3-months. There was a significant association between worksite group and change in weight at 3-months (*R*^2^ = 0.038; *F*_(3,213)_ = 2.79, *p* = 0.042) with NHHCS employees having greater weight loss than the other worksite groups (*t*_(30)_ = -3.55, *p =* 0.001). Peer educator type (internal vs. external) was also associated with weight loss (*R*^2^ = 0.020; *F*_(1,215)_ = 4.48, *p* = 0.035), such that participants with facilitators from within the worksite lost more weight than those with facilitators from outside the worksite who were brought in for this study. The number of lessons participants attended also had a significant, negative bivariate association with weight loss (*R*^2^ = 0.030, *F*_(1, 214)_ = 6.60, *p* = 0.011), such that the more lessons a participant received, the more their weight decreased.

### Multivariate analyses

Variables with a significant bivariate relationship to weight loss at 3-months and variables that differed significantly across worksite at baseline were entered into a multivariable linear model to predict weight loss at 3-month. These variables included worksite group, facilitator type, ethnicity, gender, education, baseline weight, number of lessons taken, changes in diastolic BP, physical activity frequency, fat in diet, community support, and locus of weight control. Results are presented in Table 4. The results indicated that 25.5 % of the variance is explained by the model (*R*^2^ = 0.255, *F*_(12,144)_ = 3.72, *p* < 0.001). Changes in the following variables from baseline to 3-months significantly predicted greater weight loss at 3-months: decrease in diastolic blood pressure (*β* = 0.086, *t*_(141)_ = 3.63, *p* < 0.001), greater physical activity frequency score (*β* = 0.577, *t*_(141)_ = 3.46, *p* = 0.001), increase in perceived community support (*β* = −0.151, *t*_(141)_ = −2.15, *p* = 0.033), and a more internal locus of weight control (*β* = −0.228, *t*_(141)_ = −2.29, *p* = 0.023). Employees at a NHHCS also had significantly greater weight loss (*β* = −1.425, *t*_(141)_ = −2.38, *p* = 0.019) than employees at AI, SS, or HC. Weight at baseline (*β* = −0.018, *t*_(141)_ = −1.95, *p* = 0.053) was marginally significant predictors of weight loss at 3-months. Number of lessons was not a significant predictor of weight loss at 3-months.

**Table 4:** General linear regression model predicting weight loss at post-intervention

Variable	Estimate	Standard error	*t* value	Pr > |t|
Intercept	0.8607	1.210	0.71	0.478
Worksite				
Academic vs. SS	−0.541	0.562	−0.96	0.338
HC vs. SS	−0.760	0.524	−1.45	0.149
NHHCS vs.SS	−1.425	0.598	−2.38	0.019
External vs. internal facilitator	0.317	0.412	0.77	0.443
Hawaiian vs. other	−0.014	0.433	−0.03	0.975
Female vs. male	0.099	0.674	0.15	0.883
Less than college vs. ≥college	0.294	0.412	0.71	0.477
Baseline weight	−0.018	0.009	−1.95	0.053
Change in diastolic blood pressure (mmHg)	0.086	0.024	3.63	<0.001
Change in physical activity frequency Score	0.577	0.167	3.46	<0.001
Change in community support	−0.151	0.070	−2.15	0.033
Change in locus of weight control	−0.228	0.099	−2.29	0.023

For overall model fitting, *R* ^2^ = 0.265, *F*(_12,144_) = 4.36, *p* < 0.0001

Baseline weight variables were first transformed by grand mean centering before entering the general linear regression model. Pairwise comparisons were adjusted for Tukey procedure.

An intention to treat (ITT) analysis produced results similar to the completer-based model reported in Table 4. The ITT model accounts for 20.3 % of the variance in weight loss at 3-months (*R*^2^ = 0.203, *F*_(12,191)_ = 4.04, *p* < 0.001). Greater weight loss at 3-months was again significantly predicted by decrease in diastolic blood pressure (*β* = 0.069, *t*_(188)_ = 3.63, *p* < 0.001), greater physical activity frequency score (*β* = 0.522, *t*_(188)_ = 3.25, *p* = 0.001), increase in perceived community support (*β* = −0.147, *t*_(188)_ = −2.10, *p* = 0.037), and a more internal locus of weight control (*β* = −0.220, *t*_(188)_ = −2.21, *p* = 0.028). Employees at a NHHCS had significantly greater weight loss (*β* = −1.427, *t*_(188)_ = −2.70, *p* = 0.008) than employees at AI, SS, or HC.

## DISCUSSION

Translation of evidence-based health promotion interventions into worksites was an unmet Healthy People 2010 goal and continues to be a Healthy People 2020 goal. The translation of DPP-LI to worksites, especially those with employees from high-risk groups, addresses this goal of Healthy People 2020. Translated from the evidence-based DPP-LI, the PILI@Work weight loss program was adapted for delivery to employees at various worksites of Native Hawaiian-serving organizations. These worksites included academic institutions, social service agencies, health centers, and sites within the Native Hawaiian Health Care System. Twenty-two distinct employee groups across 15 worksites completed the 3-month intervention from 2010 to 2014.

Overall, PILI@Work participants lost an average of 1.2 kg, which coincided with significant improvements in fat intake and eating self-efficacy. Albeit the overall weight loss was modest, it may have positive clinical implications for the participants. For example, DPP researchers found that every kilogram of weight loss was associated with a 13 % reduction in an individual’s risk of developing type 2 diabetes [44]. In 2012, individuals diagnosed with diabetes each incurred an average direct medical expenditure of $13,700, which is approximately 2.3 times higher than for persons without diabetes. Thus, worksite interventions, such as the PILI@Work Program, may lead to health care savings from a reduction in diabetes-related expenses. For employers, a decrease in diabetes incidence can lead to a decrease in indirect costs due to less absenteeism, increased productivity, and less short-term disability [45].

PILI@Work participants also had significant improvements in systolic and diastolic BP, consistent with other weight loss interventions [46]. Hypertension is a major risk factor for cardiovascular disease, which is the leading cause of death in the U.S. Preventing or treating hypertension, non-pharmacologically, through healthy lifestyle interventions is important in lowering the risk of cardiovascular disease. Significant improvements were also seen in physical functioning and physical activity frequency, which have been shown to be important to maintaining a healthy weight. For example, a prospective cohort study of over 400,000 people demonstrated that even small amounts of physical activity (i.e., 92 min per week or 15 min per day) resulted in a 14 % reduced risk of mortality and a 3-year longer life expectancy [47].

The categorization into four worksite groups was a post hoc decision based on the type of services provided by each organization and, using this categorization, significant differences in outcomes were observed. Employees at the NHHCS and HCs achieved greater weight loss than the other two worksites groups. Only employees at SS had significant improvement in systolic and diastolic blood pressure. And only AI employees decreased their perceptions of community support for healthy lifestyles. There are several possible explanations for these differences. AI may have a more clinically focused approach to health vs. NHHCS and HC’s, which may have a more holistic approach to health. This difference in approach may translate to a community-based, holistic approach to employee health, which is more compatible with the PILI@Work intervention. For instance, the NHHCS employees had access to an onsite fitness center, which was available to both employees and their clients. Future research should be directed at better understanding worksite factors that may impact intervention effectiveness.

An interesting and unexpected finding was that perceived community support decreased from baseline to 3-month assessment, particularly among the AI employees. While we do not have detailed information about why this perception decreased overtime, it is hypothesized that recommendations provided as part of the intervention (i.e., healthy eating and increased physical activity) inadvertently increased awareness of the lack of available options at the worksite and in the broader community. Therefore, this decrease in perceived community support for healthy lifestyles may reflect a change in the participants’ awareness of what community support entails rather than an actual decrease in that support. Future research clarifying perceived, actual, and utilized community support for healthy lifestyle choices can help to explain the changes observed in this study.

There are several limitations through which this study should be understood. This study used a pretest-posttest design. Without a randomized control trial (RCT) design, it is difficult to assess whether the changes observed were due to the intervention or other factors. However, the intervention was translated from the DPP-LI, which was already found efficacious via a RCT. Additionally, the intent of this study was to test its effectiveness when applied in real-world settings. The worksite setting where the intervention was implemented spanned six of the eight major islands of the Hawaiian archipelago (i.e., O‘ahu (9), Maui (1), Lāna‘i (1), Moloka‘i (2), Kaua‘i (2), and Hawai‘i Island (2)). Thus, we are confident that site-specific factors (e.g., a community health improvement campaign outside the worksite) did not confound our overall outcomes. There were no differences in the effectiveness of the intervention across ethnic groups. This is in agreement with a previous manuscript, which reported a lack of significant differences in weight loss by ethnicity from the PLP delivered in community settings [25]. The disproportionate number for females who enrolled in the study may have hindered our ability to detect gender differences in outcomes. Additionally, several of the factors found to change significantly over the course of the intervention are self-report. Thus, the changes reported may reflect increased awareness of the concept being measured over time (e.g., physical activity frequency, fat in diet). Finally, the participants were self-selected and, therefore, possibly more motivated to make a change in their behavior than the average employee. This may limit the generalizability of results on program effectiveness for participants who are not contemplating weight loss on their own.

### Implications

The findings of this study have several important clinical, research, and policy implications. The PILI@Work intervention was a successful translation of a community-based intervention into a worksite. This supports the assertion that the worksite is a viable venue for reaching high-risk groups to improve weight and blood pressure and ultimately decrease obesity-related mortality and morbidity. Additionally, results suggest that working with patients or client to increase their physical activity frequency and adopt a more internal locus of weight control can improve their ability to lose weight.

This study’s findings suggest that the PILI@Work intervention is more effective in Native Hawaiian Health Care Systems and community health centers, compared to large academic/medical institutions and social service organizations. More research is needed to examine specific worksite factors (e.g., wellness policies, administration support, employee characteristics) in NHHCS and HC that resulted in greater weight loss. Additionally, future research should focus creating worksite-based healthy lifestyle programs that are specifically targeted at AI and SS. Future studies may benefit from the inclusion measures of program utilization to determine dose effects.

This study also has implications for worksite wellness policies. There was an association between internal vs. external facilitator and weight loss, with internal facilitators having participants experience greater weight loss. While this association did not remain significant in a multivariate analysis, it does suggest that facilitators who are from the employee rank may be more effective in helping other employees achieve their healthy lifestyle goals. They may be better able to relate to the challenges faced by the employees at work and at home as to make the lessons more relevant. Lastly, employer sponsored health programs, which directly (e.g., provision of wellness services) or indirectly (e.g., time for physical activity during the work day) support healthy lifestyles, could result in significant cost savings for the employers and improved health for employees. With the emphasis on prevention and workplace wellness in the Affordable Care Act, there has been increase attention given to the implementation of worksite wellness programs. The PILI @ Work project illustrates that a culturally adapted, healthy lifestyle curriculum can be successfully implemented in worksites and contribute weight loss and improved blood pressure and physical functioning in participant employees.
